# A Series of Remote Melatonin Supplement Interventions for Poor Sleep: Protocol for a Feasibility Pilot Study for a Series of Personalized (N-of-1) Trials

**DOI:** 10.2196/45313

**Published:** 2023-08-03

**Authors:** Mark Butler, Stefani D’Angelo, Alexandra Perrin, Jordyn Rodillas, Danielle Miller, Lindsay Arader, Thevaa Chandereng, Ying Kuen Cheung, Ari Shechter, Karina W Davidson

**Affiliations:** 1 Institute of Health System Science Feinstein Institutes for Medical Research, Northwell Health Manhasset, NY United States; 2 St John’s University New York, NY United States; 3 Mailman School of Public Health Columbia University New York, NY United States; 4 Columbia University Irving Medical Center New York, NY United States; 5 Donald and Barbara Zucker School of Medicine at Hofstra/Northwell Health Hempstead, NY United States

**Keywords:** feasibility, insomnia, melatonin, N-of-1, personalized trial, personalized, placebo, poor sleep, sleep duration, sleep quality, supplements, virtual

## Abstract

**Background:**

Poor sleep, defined as short-duration or poor-quality sleep, is a frequently reported condition with many deleterious effects including poorer cognitive functioning, increased accidents, and poorer health. Melatonin has been shown to be an efficacious treatment to manage symptoms of poor sleep. However, the treatment effects of melatonin on sleep can vary greatly between participants. Personalized, or N-of-1, trial designs represent a method for identifying the best treatment for individual participants. Although using N-of-1 trials of melatonin to treat poor sleep is possible, the feasibility, acceptability, and effectiveness of N-of-1 trials using melatonin are unknown. Using the National Institutes of Health Stage Model for Behavioral Intervention Development, a stage IB (intervention refinement, modification, and adaptation and pilot testing) design appeared to be needed to address these feasibility questions.

**Objective:**

This trial series evaluates the feasibility, acceptability, and effectiveness of a series of personalized interventions for remote delivery of melatonin dose (3 and 0.5 mg) versus placebo supplements for self-reported poor sleep among 60 participants. The goal of this study is to provide valuable information about implementing remote N-of-1 randomized controlled trials to improve poor sleep.

**Methods:**

Participants will complete a 2-week baseline followed by six 2-week alternating intervention periods of 3 mg of melatonin, 0.5 mg of melatonin, and placebo. Participants will be randomly assigned to 2 intervention orders. The feasibility and acceptability of the personalized trial approach will be determined with participants’ ratings of usability and satisfaction with the remote, personalized intervention delivery system. The effectiveness of the intervention will be measured using participants’ self-reported sleep quality and duration and Fitbit tracker–measured sleep duration and efficiency. Additional measures will include ecological momentary assessment measures of fatigue, stress, pain, mood, concentration, and confidence as well as measures of participant adherence to the intervention, use of the Fitbit tracker, and survey data collection.

**Results:**

As of the submission of this protocol, recruitment for this National Institutes of Health stage IB personalized trial series is approximately 78.3% complete (47/60). We expect recruitment and data collection to be finalized by June 2023.

**Conclusions:**

Evaluating the feasibility, acceptability, and effectiveness of a series of personalized interventions of melatonin will address the longer term aim of this program of research—is integrating N-of-1 trials useful patient care? The personalized trial series results will be published in a peer-reviewed journal and will follow the CONSORT (Consolidated Standards of Reporting Trials) extension for N-of-1 trials (CENT 2015) reporting guidelines. This trial series was approved by the Northwell Health institutional review board.

**Trial Registration:**

ClinicalTrials.gov NCT05349188; https://www.clinicaltrials.gov/study/NCT05349188

**International Registered Report Identifier (IRRID):**

DERR1-10.2196/45313

## Introduction

Sleep health is understood to be a multidimensional construct influenced by dimensions such as duration, satisfaction, timing, efficiency, and alertness, and it is a partially subjective experience that should be assessed in the context of the individual. In recent years [1], the prevalence of self-reported difficulty sleeping has increased, and population studies have shown increasing trends toward shorter sleep duration [2]. A recent study of US workers found the prevalence of short sleep duration to be 37.6% and associated poor sleep quality to be 19.2% [3]. Deleterious effects of poor sleep include a heightened stress response; pain; depression; anxiety; and deficits in cognition, memory, and performance [4]. Short sleep duration or poor-quality sleep is also associated with health outcomes including obesity [5], diabetes [6], hypertension [7], cardiovascular disease [7], higher productivity loss [8], reduced quality of life [9], depression [9], and mortality [8]. The causes of poor sleep, defined by short sleep duration and the commonly associated poor-quality sleep [10], can be complex and diverse. These include personal factors such as sex, mental health status, and BMI [8]. Lifestyle factors such as smoking, poor diet, and low physical activity (less than 120 minutes) also predict short sleep duration [8]. Further, environmental factors including financial concerns, children, marital status, irregular work hours, and a long commute can also influence sleep quality and duration. Finally, discrete sleep disorders such as obstructive sleep apnea, insomnia, and restless leg syndrome may also affect sleep [11]. The complex etiology of sleep duration and quality means that not all individuals suffering from poor sleep have comparable presentations. The widespread and increasing nature of poor sleep and the gravity of its effects support the need for innovative treatments. Furthermore, the diversity of factors contributing to poor sleep warrants treatment that accounts for individual differences among participants. Poor sleep has been determined as high priority for a personalized trial approach by previously interviewed clinicians and patients [12].

Exogenous melatonin supplements are widely used to treat insomnia and sleep disorders as well as to adjust altered sleep schedules related to jet lag [13]. Doses of melatonin available over the counter range from 0.3 mg to a maximum of 10 mg, but they typically fall within the range of 0.3 to 3 mg [14]. Because melatonin is naturally occurring within the brain and external food sources such as fish, eggs, milk, nuts, and seeds [15], it is considered a dietary supplement, therefore warranting less regulation before distribution than a typical drug [16]. As such, research evaluating the efficacy of melatonin across populations is still developing. However, various clinical trials have been conducted demonstrating the efficacy of exogenous melatonin in treating sleep disorders regardless of the etiology of the disorder [17-20].

Functionally, endogenous melatonin secretion is governed by the suprachiasmatic nucleus (SCN) of the hypothalamus, which is the primary circadian pacemaker in mammals [21]. In addition to its endogenous activity, the SCN responds to retinal light exposure to help regulate circadian rhythms, which impact significant processes relevant to survival, including sleep, wake, appetite, mood, hormone secretions, and behaviors. During the daytime, the SCN, reinforced by external light patterns, activates processes that maintain wakefulness, attention, and cognitive capacity. Melatonin, secreted by the pineal gland primarily during darkness or nighttime in response to a signal from the SCN, is thought to facilitate sleep in part by inhibiting processes associated with wakefulness [21]. Therefore, exogenous melatonin supplements are thought to promote increased sleep duration by signaling to melatonin receptors that day has, in fact, shifted into night [22]. As such, melatonin has been found to be particularly useful in facilitating sleep initiation and increased sleep duration for individuals with disrupted circadian rhythms resulting from, for example, overnight shift work, jet lag, or sleep-wake disorders [23]. Melatonin has also been indicated in improving sleep efficacy and daytime sleepiness in healthy volunteers [24].

Importantly, clinical trials of melatonin therapy for sleep have found that not all participants benefit equally from treatment with melatonin. Several meta-analyses assessing the efficacy of melatonin therapy found evidence for significant heterogeneity in the effectiveness of melatonin interventions, particularly for outcomes including sleep onset latency, total sleep duration, and sleep efficiency [25,26]. Furthermore, the authors of a recent systematic review assessing melatonin’s effect on these sleep dimensions across 23 clinical trials identified significant between-study heterogeneity, which they attributed to differences in participant health status [27]. Participants with respiratory diseases, metabolic disorders, and sleep disorders saw greater sleep improvement from melatonin therapy than participants with a variety of other health concerns, including mental disorders, neurodegenerative diseases, and breast cancer [27]. Authors across meta-analytic publications additionally cite concerns regarding the heterogeneity of treatment dosage between studies; without consistent dosage information, the authors could not draw robust conclusions regarding overall treatment efficacy or minimum and maximum dosage recommendations for different populations [26,28]. These findings suggest that not all participants benefit equally from treatment with melatonin.

Because of this variability in treatment provision and response, participants taking melatonin may benefit from a personalized (N-of-1) trial approach [29,30]. Personalized trials are a research approach that helps participants to select which treatments work best for them individually. Participants in a personalized N-of-1 trial receive a multiple crossover trial with continuous collection of objective outcome data throughout the trial. Interventions are delivered in alternating time periods of treatment, alternative treatment, and placebo therapies, usually in randomized blocks [31,32]. At the completion of the personalized trial, the effect of each intervention is evaluated and the trial participants are able to select which intervention works best for them. Personalized trials are designed to provide patients with high-integrity, evidence-based information uniquely relevant to the outcomes and values important to them so they may identify efficacious treatments [33]. Despite the potential of personalized trials, N-of-1 personalized designs are not often used in clinical practice [31,34-36]. In surveys that examined attitudes about personalized trials, hesitancy to engage in personalized N-of-1 trials was attributed to the cost and effort required for implementation [31,34,36].

In the context of melatonin therapy, personalized trials would elucidate gaps in previous research by helping to identify the optimal melatonin treatment for participants despite a variety of demographic, health-related, and environmental factors. However, given prior issues with implementing personalized N-of-1 trials, it is important to evaluate the feasibility of this type of intervention for feasibility and acceptability from a participant perspective and the effectiveness of melatonin delivered in a personalized N-of-1 trial. On the basis of guidelines in the National Institutes of Health Stage Model for Behavioral Intervention Development [37], we will conduct a stage IB (ie, intervention refinement, modification, and adaptation and pilot testing) design trial to address these feasibility questions.

This study evaluates the feasibility, acceptability, and effectiveness of a series of personalized interventions for remote delivery of higher dose melatonin at 3 mg, lower dose melatonin at 0.5 mg, and a placebo supplement in 60 participants with self-reported poor sleep. By using new wearable technologies (such as Fitbit devices), commercially available supplements (such as melatonin produced by Pure Encapsulations), and commercially available medication adherence devices (such as Nomi by SMRxT), the current protocol allows for remote assessment of outcomes and thereby also continuous data collection. Further, remote delivery of the intervention allows each participant to receive the intervention and be assessed for poor sleep in their own home with limited disruption. Results from this study will determine whether remote delivery of these interventions is feasible and acceptable for participants with self-reported poor sleep and will allow clinicians to identify whether remote delivery of melatonin can effectively allow patients to manage their sleep.

## Methods

### Study Design

**Figure 1:**
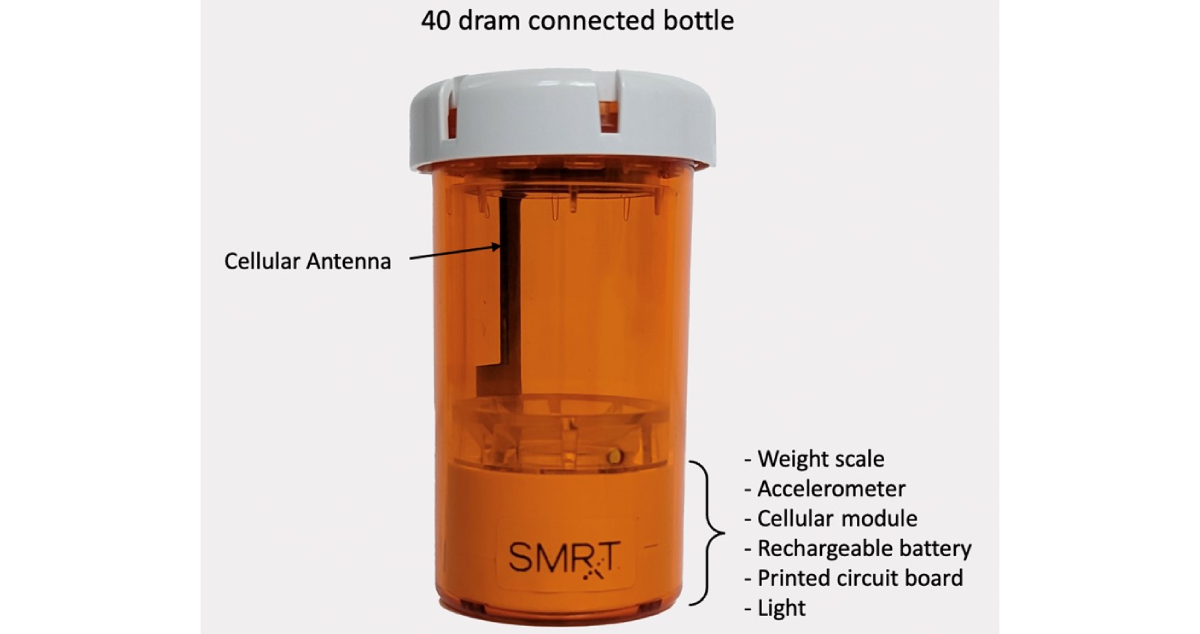
The Nomi pill bottle by SMRxT.

The study is a series of 60 randomized personalized N-of-1 trials examining the effects of 3 mg of melatonin and 0.5 mg of melatonin versus placebo for poor sleep. The intervention will be delivered remotely to participants residing in the United States over the course of 14 weeks. Participants will be provided with a Fitbit Charge 5 device, all necessary supplements, and 3 Nomi by SMRxT medication adherence devices. Nomi by SMRxT is a smart pill bottle that monitors medication-taking behavior in real time to collect adherence data without active participant input (see Figure 1).

The first 2 weeks of the study will be a baseline assessment period. Participants will not take any melatonin or placebo pills during this time. During baseline assessment, each study participant will be asked to both engage in their usual methods of managing sleep and wear their Fitbit device at all times, including during sleep. Participants will also be asked via SMS text messaging to rate an ecological momentary assessment (EMA) of their fatigue, pain, concentration, stress, mood, and confidence at 3 random times throughout the day. Each morning, participants will answer a survey questionnaire assessing their sleep quality from the previous night. Every other Sunday evening, participants will complete a longer survey measure asking them to reflect on their sleep quality over the past 2 weeks. Participants will be encouraged to wear their Fitbit devices day and night, asked to sync their device with the Fitbit application on their phone at least every 2 days, and asked to charge their Fitbit device at least every 4 days.

After successfully completing the baseline period, participants will be randomized into 2 arms with six 2-week intervention blocks of 3 mg of melatonin, 0.5 mg of melatonin, or placebo pills. During the intervention, participants will be discouraged from taking additional melatonin or using other sleep treatments outside those provided during the study. Within 3 months of the end of the 14-week trial, each participant will be provided with a report containing their analyzed data and asked to complete a satisfaction survey. After the satisfaction survey is completed, study coordinators will reach out to each participant to interview them about their experience with the personalized trial. Study recruitment began in April 2022, and the study completion is anticipated to occur by June 2023.

### Study Population

Participants in this study will be volunteer subjects from across several states within the United States. This will include the approximately 77,000 employees of the Northwell Health System [38]. Digital recruitment of potential participants will include email blasts and advertisements in newsletters circulated to the Northwell Health team member population. To expand beyond the Northwell Health system, recruitment will also target web and social media; Facebook and Google ads for the study will be published in users’ web feeds and searches. Additionally, web posts to communities on platforms such as Reddit, Craigslist, and LinkedIn will also be used to appeal to individuals with sleep issues. With 93% of US adults reporting using the internet, 69% reporting ever using Facebook, and 18% reporting using Reddit, use of digital recruitment methods significantly broadens the potential span of our outreach [39].

All potential participants will be required to self-identify as having a minimum threshold of poor sleep for at least 4 weeks using the Insomnia Symptom Questionnaire (ISQ). Poor sleep for this study was defined by self-reported difficulty in falling asleep, staying asleep, and feeling refreshed by sleeping 4 to 7 times per week for a period of at least 4 weeks. Participants must also report 1 or more sleep symptoms that bother them “quite a bit” or “extremely.” Potential sleep symptoms on the ISQ include disrupted work, irritability, and feeling fatigued. An example of the ISQ measure used to assess sleep disruption can be found in Multimedia Appendix 1. Because of the high prevalence of poor sleep [3] and wide reach of our recruitment methods [39], we anticipate a large pool of potential participants from which to recruit.

### Recruitment and Consent

Participants for this study will be recruited via advertising and social media posting on Facebook, Instagram, Google, Craigslist, LinkedIn, and Reddit. Posting and advertisement will comprise multiple formats of information (including videos, images, and text posts). Additional recruitment methods will include Northwell Health team member email listserves, word of mouth, recruitment emails to individuals who previously expressed interest in personalized trials, and the Northwell Health Clinical Trials Listing.

Interested individuals who respond to any of the above recruitment methods will be directed to an digital information screen with details about the pilot study and asked to complete screening measures containing questions regarding study inclusion and exclusion criteria (Textbox 1). This information will be reviewed by study staff to determine participant eligibility before consent. If a potential participant is deemed ineligible or is wait-listed due to high demand, study staff will reach out to the participant to notify them within 2 business days. Upon completion of screening, eligible individuals will be provided with a link to an electronic consent form and a short video explaining details of the study protocol and consent form. If individuals wish to speak with a staff member regarding details of the study or consent, they will be offered a 30-minute informational phone call with study staff to review key points within the consent form and any questions the potential participant has about the study. A study staff member will confirm the scheduled time for this call with the participant within 2 business days. After the phone call, the study staff will send the eligible participant a message containing the electronic consent form, the explanatory video, and a 4-question screening measure assessing participant understanding of the protocol and consent process.

**Inclusion and exclusion criteria.**

**Inclusion criteria**

- Aged ≥18 years
- Fluent in English
- Able to take melatonin and a placebo
- Self-report of poor sleep symptoms using the Insomnia Symptom Questionnaire
- Own and can regularly access a smartphone capable of receiving SMS text messages
- Owns and can regularly access an email account
- Lives in the United States
- Agrees to adhere to lifestyle considerations including wearing a Fitbit device day and night and potentially adapting their current melatonin routine to fit the protocol throughout the study duration

**Exclusion criteria**

- Aged <18 years
- Pregnant or breastfeeding
- Diagnosed with depression, seasonal affective disorder, schizophrenia, autoimmune disease, or asthma
- Take monoamine oxidase inhibitors or corticosteroids
- Diagnosed with low blood pressure
- Clinically diagnosed with a sleep disorder (eg, narcolepsy, circadian rhythm sleep-wake disorders, periodic limb movement disorder, restless leg syndrome, obstructive sleep apnea, etc) 
- Deemed unable to complete the study protocol due to cognitive impairment, severe medical or mental illness, or active or prior substance abuse 
- Participate in shift work (evening/night shifts, early morning shifts, rotating shifts, etc) 
- Frequently travel across time zones (eg, pilots or flight attendants)
- Receive specialty mental health care for insomnia (eg, cognitive behavioral therapy for insomnia and medications for insomnia) 
- Advised by a doctor or health care provider that taking melatonin is unsafe
- Do not own or cannot regularly access a smartphone capable of receiving SMS text messages
- Do not possess or cannot regularly access an email account
- Lives outside of the United States
- Scheduled for planned surgery within 6 months from the study start date

Persons who are eligible to participate after screening and who opt out of the educational phone call will receive a message from study staff containing an electronic copy of the consent form and an explanatory video. These potential participants will also receive the same 4-question screening measure to assess their understanding of the consent form and study protocol.

Consent will be obtained electronically. Participants will receive a copy of the institutional review board (IRB)–approved consent form, a copy of their signed consent form, and a copy of their signed HIPAA (Health Insurance Portability and Accountability Act) authorization form via an encrypted message. Signed consent and HIPAA authorization forms will be stored electronically on a HIPAA-compliant, Northwell Health–approved shared drive accessible only to the IRB-approved study staff. The example study consent form can be found in Multimedia Appendix 2.

After consent, participants will be able to choose a study start date during their enrollment process from a provided list of dates. No more than 20 potential participants will begin their baseline period on the same day. Study enrollment will continue until 60 participants have been randomized after the baseline assessment.

### Assignment of Interventions

**Figure 2:**
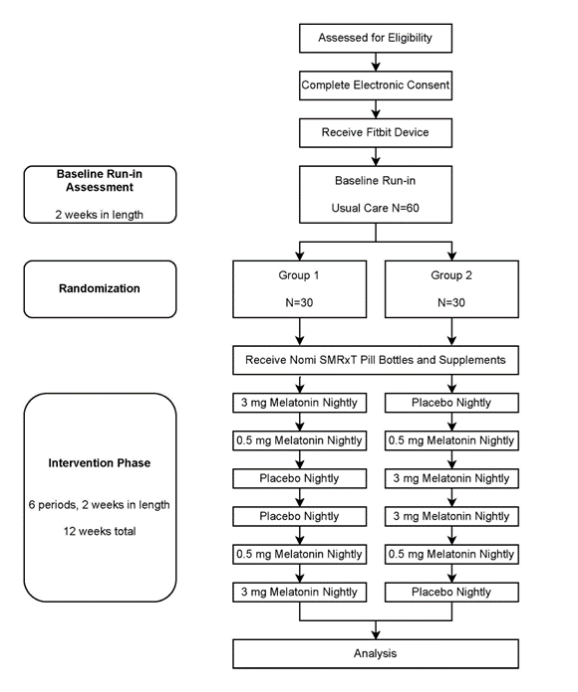
Participant Timeline.

Each treatment was assigned a letter with A=3 mg of melatonin, B=0.5 mg of melatonin, and C=placebo. Of those participants who are enrolled in the study, approximately 30 will be randomized by the study statistician to receive the protocol in the following order of 2-week treatment periods: 3 mg of melatonin, 0.5 mg of melatonin, placebo, placebo, 0.5 mg of melatonin, 3 mg of melatonin (ABCCBA). Approximately 30 more participants will be randomized in the following order of 2-week treatment periods: placebo, 0.5 mg of melatonin, 3 mg of melatonin, 3 mg of melatonin, 0.5 mg of melatonin, placebo (CBAABC). This randomization pattern can be viewed in the participant time line in Figure 2.

### Interventions

Once a participant successfully completes baseline and is randomized into the study, they will be mailed 3 Nomi pill bottles (see Figure 1). Each bottle will contain 28 pills of 3 mg of melatonin, 0.5 mg of melatonin, or placebo labeled “A,” “B,” or “C.” The doses of 3 and 0.5 mg of melatonin were selected to compare high and low doses of melatonin, respectively. Previous studies have classified doses of 0.3 to 1 mg as low doses of melatonin, which may still be effective [40]. Higher doses of melatonin (ranging between 2 mg and 6 mg) have been found to be effective for treating sleep disruption [41,42]. In this study, 3 and 0.5 mg of melatonin were selected because they are provided by the manufacturer, and color- and size-matched placebos are available. Both the 3 and 0.5 mg of melatonin pills are presented in the same type of capsule (ie, same size and appearance) but with different concentrations of melatonin. The placebo pill is an identical capsule filled with a glucose solution. The melatonin and placebo doses provided for this study are prepared by Pure Encapsulations LLC, a National Sanitation Foundation Good Manufacturing Practices–registered laboratory that exceeds United States Pharmacopeia standards for supplement manufacturing. This laboratory has a 50-year history of producing products for clinical research [43-45]. Participants will receive a treatment schedule that indicates which pill bottle of medications is assigned to each week of the intervention. During intervention weeks, participants will receive nightly SMS text message reminders an hour before their self-reported bedtime, instructing them to take a pill from their assigned pill bottle depending on where they are in the randomization time line. Participants will be asked to refrain from taking additional melatonin for the duration of the study. During all treatment periods, participants will be asked to wear the Fitbit device 24 hours a day and answer up to 5 survey measures sent to them via SMS text messaging daily.

### Participant Time Line

Figure 2 illustrates the participant time line.

### Adherence

During the baseline period, the study team will assess participant adherence to the protocol. Adherence will be evaluated using the Fitbit tracker and survey completion. Participants wearing the Fitbit for 12 or more hours per day, including at least 180 minutes of sleep time, will be defined as adherent [46,47]. During the 14 days of the baseline period, participants who do not achieve a minimum of 80% adherence to Fitbit tracker wear and study measures (including EMA and survey measures) will be withdrawn from the study before randomization. Those participants with 80% adherence or higher by the final day of the baseline period will be randomized to the intervention phase. To encourage adherence, participants will be provided with short educational videos and protocol reminders via SMS text messaging, and they will be encouraged to contact study staff with concerns by phone, email, or secure portal message.

### Participant Report

Participants who complete the intervention portion of the trial will have generated a 15-page personalized report summarizing their trial results and showing the impact of 3 mg of melatonin, 0.5 mg of melatonin, and placebo on their Fitbit-recorded sleep duration. The goal of the report is to display participants’ sleep duration averaged across each study intervention with the goal of helping participants to select which treatment works best for them. The participant report also presents summaries and results for other sleep- and wellness-related outcomes that are both Fitbit-recorded (such as sleep efficiency, average time spent in sleep stages, and average number of steps) and self-reported (such as daily sleep quality and EMA fatigue). This report is not to be interpreted as medical advice. Participants are encouraged to seek a medical professional’s opinion if they have any questions or concerns about the data displayed in their report. A similar version of the participant report in a series of personalized trials for chronic lower back pain is described and displayed in further detail elsewhere [48].

Results for the participant report are generated by comparing the effectiveness of 3 mg of melatonin and 0.5 mg of melatonin relative to placebo for each of the outcomes measured in the study. The participant report contains information about the magnitude of the difference for each outcome between each treatment period. Participants will be provided information about whether the difference was statistically significant (defined as α≤.05) in generalized least-squares (GLS) regression analyses. They will also be provided with estimates of their adherence to the intervention, Fitbit device, and survey measures.

After automated generation by the study data team, each participant report will be individually reviewed for accuracy, consistency, and comprehensibility by several study team members before delivering the report to the participant as a printable PDF file via a secure web portal. Personalized participant reports will be delivered to the participants within 3 months of their intervention completion, and they will be given the option to review their report along with a study team member. Participants will have an opportunity to provide their feedback on their report in the satisfaction survey and follow-up study interview.

### Patient and Public Involvement Statement

Pilot data with participants and internal use testing were used to help determine which medication adherence device to select for this trial. We did not directly involve participants in any other elements of the design of this trial.

### Ethics Approval

This study underwent ethics review and was approved by the Northwell Health IRB (IRB #21-0239). All amendments to the protocol will be submitted for approval to the ethics committee and Northwell Health IRB.

### Data Monitoring

A Data and Safety Monitoring Board (DSMB) will be assigned to periodically assess for participant safety, scientific integrity, and the trial’s progress. This DSMB will include 4 members who will have varying areas of scientific expertise including statistics and medicine. The DSMB conduct periodic reviews of study data for accuracy, completeness, and timeliness of submission. The study DSMB will also assess for evidence of potential harms, including adverse treatment events and loss of confidentiality. Following these reviews, the DSMB will be responsible for making recommendations regarding the continuation, modification, and termination of the project in reports provided to the study principal investigator. Regular collaboration between the study team and the DSMB will ensure access to study data for monitoring and regulatory inspection.

### Treatment Adverse Events

Melatonin poses a low risk of physical harm to subjects, as it has a very low side effect profile and limited evidence of habituation and tolerance. A 2015 review on the safety of melatonin supplements indicated that only mild side effects were reported in various short-term studies that involved adults, surgical patients, and critically ill patients [49]. Some of the mild side effects that were reported in the studies included headache, dizziness, nausea, and sleepiness [49]. Less common side effects include short-lasting feelings of depression, mild tremor, mild anxiety, abdominal cramps, irritability, reduced alertness, confusion or disorientation, and abnormally low blood pressure. The chances that participants could experience these side effects during the trial are low given the duration and dosing proposed for this research [50]. Participants document any side effects they experience that may be related to the supplements to the study team daily in the morning survey. Participants are informed that they may discontinue supplement dosing at any point during the trial.

The exclusion criteria for this study were used to prevent participants who are at greater risk of harm from participating in the study. Protocol adherence is monitored throughout the trial, and participant re-education is conducted as needed.

### Loss of Confidentiality or Privacy

A risk that may be incurred by participating in the trial is a loss of privacy or confidentiality. The participant is made aware of all the data collected and the companies and devices employed to collect the data via the consent process. All identifying information of participants is stored in a secure, password-protected, Northwell Health–approved, HIPAA-complaint database, and personal and identifying information is not stored on any devices used in the study. Using the devices employed in this trial as part of this study as compared with using the devices as a consumer presents no additional risk. Once participants complete their study involvement, identifying information will be destroyed. All research team members with access to identifiable and deidentified data are trained and included on the IRB submission for approval. Regular meetings will occur with the principal investigator and other study team members to ensure protocol adherence and data accuracy.

### Costs

This pilot study is funded by the National Institutes for Health (R01LM012836). All study-related equipment, devices, procedures, melatonin, and placebo supplements will be provided to participants at no cost, and participant insurance will not be billed. This study uses SMS text messaging to deliver notifications, reminders, and study questionnaires, which may include standard message and data rates from the participants’ wireless carrier. Study participants will not be compensated for any costs related to data usage or sending or receiving SMS text messages by the study or by members of the study team.

### Compensation

After completing all components of the study (ie, successful completion of a research team–approved intervention period, submission of a satisfaction survey, and returning the Nomi devices), study participants will be mailed a US$ 100 payment card (ClinCard). Additionally, as a thanks to study participants for their participation, they will be allowed to keep their Fitbit Charge 5 (a value of US$ 150.00).

### Primary Outcome

The primary outcomes of this study will be feasibility as measured by the mean usability score evaluated using the 10-item System Usability Scale (SUS) [51] and acceptability as measured by an 18-item satisfaction survey. The SUS is a validated questionnaire that asks users to score each item on a Likert scale from “strongly disagree” (1) to “strongly agree” (5) [52,53]. Individual item scores are multiplied by 2.5 and summed to generate a total score ranging from 0 to 100, with higher scores indicating a greater level of usability. This measure has been used and validated in multiple contexts [52,53]. The SUS will be presented to the participants as addressing the ease of use, complexity, and consistency of the personalized trial system as a whole. Satisfaction will be measured using a 6-item satisfaction survey. The survey will assess participant satisfaction with elements of the trial, including explanatory resources, the Fitbit device, the personalized trial design, survey assessment measures, and the participant report. Participants will be asked to rate their satisfaction on a scale of 1 (“not very satisfied”) to 5 (“very satisfied”).

The satisfaction survey will also include a 7-item series of questions regarding the participant’s experience in the personalized trial as a whole. Satisfaction with aspects of the trial including the onboarding process and ease of trial adoption will be rated on a scale of 1 (“strongly disagree”) to 5 (“strongly agree”). Finally, satisfaction with the personalized report will be assessed through 5 questions on a scale of 1 (“strongly disagree”) to 5 (“strongly agree”).

### Secondary Outcomes

The secondary outcomes in this study will include Fitbit device–recorded nightly sleep duration, self-reported sleep quality using a modified version of the Consensus Sleep Diary (CSD), EMA self-reported fatigue ratings, EMA self-reported pain ratings, adherence to use of the Fitbit device, and adherence to each of the interventions (3 mg of melatonin, 0.5 mg of melatonin, and placebo).

Nightly sleep duration and daily steps will be assessed using non–Near Field Communication, Fitbit Charge 5 devices. During baseline assessment (2 weeks) and all intervention weeks (12 weeks), participants will be asked to wear their Fitbit device each day and night (for a total of 14 weeks overall). All enrolled participants will be provided with a Fitbit device and Fitbit account that the research team has created with no identifying information for use in the study. A file linking the Fitbit account identifier to the study participant will be housed in a Northwell Health–approved drive to store protected health information. It will be accessible solely to members of the study team listed in the IRB application. Participant Fitbit data will be retrieved using N1Thrive and Fitabase, both secure web portals. Participants’ study Fitbit accounts will be linked to an identification number in the N1Thrive and Fitabase systems. No identifying information will be stored in the Fitabase data set. Fitabase and N1Thrive will stop tracking participant Fitbit data at the end of the trial. As an added security measure, participants will be instructed to remove the Fitbit study account from their smartphone device to keep the Fitbit.

The CSD is a daily instrument used to assess and track intervention effects on sleep symptoms, quality, and satisfaction [54]. Three versions of the CSD were originally put out by its authors: the core CSD containing 9 items, an expanded morning version with optional additional items to be completed upon arising, and an expanded evening version with separate morning and evening items to be optionally completed. This study uses a modified version of the core CSD, asking participants about perceived sleep quality (via a Likert scale), sleep onset latency, the time at which the participant believes sleep onset occurred, duration of nighttime awakenings, final rise time, and a multiple-selection question asking if any of 10 (or an optional “other” selection) listed factors directly impacted the participant’s sleep. The CSD will be sent to participants within an hour of their self-reported wake time each day of the study to assess and monitor patient-reported outcomes regarding sleep quality throughout each intervention period of the trial. Data observed through the CSD will be reported back to participants via the personalized participant report.

Daily self-reported fatigue and pain ratings will be assessed via EMA using a measure adapted from the Numeric Pain Rating Scale [55]. These assessment measures are single-item assessments administered 3 times daily via SMS text messaging asking participants to rate their fatigue and pain in the current moment on a scale of 0 to 10. The timing of the SMS text messages will be randomized between a participant’s self-reported wake and sleep times. For fatigue, ratings of 0 indicate no feeling of fatigue, with scores of 1 to 3, 4 to 6, 7 to 9, and 10 respectively indicating a little, somewhat, quite a bit, and very much feeling fatigued. Interpretations of scores remain the same for pain. EMAs are collected daily via surveys using the secure study platform N1Thrive, a Northwell Health–approved and HIPAA-compliant system used for patient engagement and collecting and storing research data. A workflow was constructed for this study to include automated messaging pathways delivered via SMS text messaging directly to the participant’s smartphone via the platform.

Participant adherence to the intervention and time of melatonin or placebo use will be measured using the Nomi smart pill bottle. The Nomi system includes a smart bottle that is designed to look just like a typical prescription pill bottle. The bottle comes equipped with a sensor that measures patient adherence using weight, time, movement, and temperature. The data are then made available to health care providers via a cellular connection to the SMRxT cloud. Below the sensor is a light that flashes when it recognizes that a dose has been taken, the device’s battery is low, or the device is unable to connect to the network. From the digital Nomi dashboard, study team members will remotely assign treatments to patients, create treatment schedules, and monitor medication usage. No patient information or identifying data will be stored on the device or within the Nomi adherence dashboard—only unique study IDs will be used to collect data. The bottle serial number and the unique study ID will be linked to subjects in REDCap, a secure data management system managed only by research staff. The Nomi system will also be integrated with the study platform to deliver automated SMS text message reminders in cases of deviation from assigned supplement dosing.

### Exploratory Outcomes

The exploratory outcomes in this trial will include self-reported sleep disturbance measured using the Insomnia Severity Index (ISI), participant self-reported side effects, EMA self-reported pain ratings, EMA self-reported mood ratings, EMA self-reported concentration ratings, and EMA self-reported confidence ratings. We will also measure participant adherence to survey measures and EMA assessment measures. The ISI is a brief patient-reported outcome insomnia instrument assessing the severity of nighttime insomnia and related daytime impairment [56]. It comprises 7 items, each scored using a 5-point Likert scale, which summate to a total score. Lower ratings on the scale equate to less disturbance (0=no problem) and higher ratings of more disturbance (4=very severe). This overall score can function as a diagnostic of the severity of clinically significant insomnia or subclinical sleep disturbances. The ISI will be sent to participants as a biweekly survey every other Sunday evening (ie, at the end of baseline and the end of each intervention block), and it will ask them to reflect on their sleep disturbance symptoms for the past 2 weeks (or the past study block). This instrument will therefore be the key data point to solicit participants’ self-reported sleep disturbance on a retrospective basis for an intervention period as a whole. ISI data will be reported back to the participants in the participant report.

Patient self-reported side effects will be assessed daily using a survey measure. EMA measures of stress, mood, confidence, and concentration will be assessed in using single-item assessments administered 3 times daily via SMS text messaging asking participants to rate how they feel in the current moment on a scale of 0 to 10. Delivery and assessment of these measures will be identical to the methods used for EMA fatigue and pain described above. Participant adherence to survey measures (including the sleep diary and ISI) and EMA check-in measures will also be tracked.

## Results

### Recruitment Status

As of the submission of this protocol, recruitment for this pilot is approximately 78.3% (47/60). We expect recruitment and data collection to be finalized by June of 2023.

### Sample Size Calculation

The sample size of 60 participants was chosen to ensure a sufficient number to obtain a preliminary assessment of the feasibility of this series of personalized trials of melatonin therapy for sleep. The feasibility for this study is determined using scores from the SUS, which is administered at the completion of the trial. With 60 participants receiving the intervention and use of a single sample binomial test with α set to 2.5% significance, we will have approximately 90% power if 70% of participants complete the trial. With 60 randomized participants—and an expected trial completion rate of 70%—we anticipate SUS data to be available in approximately 42 participants, thus giving a standard error no greater than 8% in estimating the rate of SUS ≥85, an exceptional level of usability [57]. Individual participant responses will be reported so that individual-level heterogeneity can be assessed and evaluated [58].

### Primary Analysis

The primary analysis will be based on usability ratings submitted by all enrolled participants (n=60) on the SUS. These usability ratings will be summarized using descriptive statistics including means, medians, SDs, and IQRs and visualized via a histogram. If the trial receives a score of 70 or greater, it will be judged to have “acceptable” levels of feasibility, whereas scores of 80 or greater are interpreted as “excellent” [57]. The SUS has been used and validated for use in multiple programs and trials [52,53].

We will also analyze participant self-reported levels of satisfaction. Self-reported satisfaction levels will be reported for each component of the trial (eg, interventions and design) using means and SDs. Participant responses to each satisfaction item will also be depicted graphically to show the distribution of participant responses from “not very satisfied” to “very satisfied.” This satisfaction measure has previously been used to evaluate acceptability of personalized trials [47].

### Secondary Analyses

Means and SDs for Fitbit device–recorded nightly sleep duration, self-reported sleep quality using a modified version of the CSD, EMA self-reported fatigue ratings, and EMA self-reported pain ratings will be reported for the baseline assessment period (2 weeks) and each intervention period (6 blocks of 2 weeks) and depicted graphically. Proportion of days adherent to the Fitbit device and adherence to the intervention will be reported using mean proportions and SDs for the study overall. We will also calculate mean and SD values for each secondary outcome across intervention periods for 3 mg, 0.5 mg, and placebo. For example, we will sum all outcome measures for both the 2-week intervention periods to derive an overall mean value for the 3 mg melatonin period. The same process will be followed for the 0.5 mg melatonin and placebo periods. As the timing of melatonin doses may influence the effect of melatonin on sleep [59-61], the time of dose relative to sleep onset will be examined in sensitivity analyses.

Finally, the effects of each intervention (3 mg of melatonin, 0.5 mg of melatonin, and placebo) on the secondary outcomes will be examined using generalized linear mixed models with an autoregressive, AR(1), model. This model accounts for possible autocorrelation and linear trends between fatigue ratings across time. We will consider week as a linear term and factor in the mixed model to explore the nonlinear time effects of each treatment. This will generate estimates of each intervention on each of the secondary outcomes for the overall sample. In addition, we will conduct analyses to evaluate the effects of each intervention on the secondary outcomes at the level of the individual participant. Using GLS analyses with an AR(1) model, we will be able to generate estimates of the effect for each intervention for each participant for each of the secondary outcomes. This level of individual-level analyses is essential for personalized trials.

### Exploratory Analyses

Exploratory outcome measures, including ISI, participant self-reported side effects, EMA self-reported pain ratings, EMA self-reported mood ratings, EMA self-reported concentration ratings, and EMA self-reported confidence ratings, will be reported using means and SDs overall and by the study period. Similar to analyses of secondary outcomes, the effects of each intervention on the exploratory outcomes will be evaluated with generalized linear mixed model analyses using autoregressive models in the overall sample and with GLS analyses using autoregressive models with individual participants. Exploratory adherence measures (adherence to surveys and EMA measures) will be represented using proportions for the overall study and by the study period. Comparisons between the 2 treatment orders (ABCCBA and CBAABC) will also be conducted to identify potential confounding effects due to treatment order.

## Discussion

This study represents an advancement in the process of developing feasible, acceptable, and effective personalized interventions for a variety of health conditions. Widespread use of personalized N-of-1 designs has been hampered by difficulties in the feasibility of creating them, with many researchers and clinicians feeling that personalized N-of-1 trials are not the worth the effort needed to conduct them [62]. In assessing the feasibility and acceptability of our developing personalized N-of-1 platform, which has been previously implemented to treat chronic lower back pain [47] and fatigue [46], we hope to continue refining a programmatic template for using personalized N-of-1 designs for other health conditions and other interventions. Further, we hope the use of digital enrollment, consent, intervention delivery, and outcome assessment will make personalized N-of-1 trials more feasible in research and clinical practice.

Potential limitations for the current trial include potential issues with representativeness of the participant sample and with doses of melatonin selected in the trial. Although our recruitment efforts are directed to identify a diverse sample for enrollment in the current trial, inaccurate sampling of participants with disrupted sleep is possible. For example, using digital recruitment methods may oversample individuals who have disrupted sleep due to excess internet usage time or exposure to blue light. For evaluations of effectiveness, this is not a concern since the goal of a personalized N-of-1 trial is to assess treatment effectiveness in each individual participant. However, the primary goal of this trial is to evaluate the feasibility and acceptability of personalized trials for poor sleep. Failing to recruit a representative sample of participants with poor sleep may limit our conclusions regarding the feasibility and acceptability of personalized N-of-1 trials of melatonin for sleep. In addition, individual participants may respond more optimally to other doses of melatonin than those examined in the current trial (namely 0.5 and 3 mg). Although these doses are representative of lower and higher doses of melatonin used in previous sleep interventions, assuming that some individuals may respond optimally to higher doses of melatonin (eg, 6 mg) or to doses not examined in the current trial (eg, 1 mg) is reasonable. In addition, some research suggests that depending on how melatonin supplements are prepared, the dose within each supplement may also vary [63]. Further, it is possible that dose duration may influence findings, as longer duration melatonin treatments have been associated with increased sleep duration [25]. For example, a participant may have responded better to a longer duration (eg, a 4-week block of melatonin rather than a 2-week block). However, the current goal of this trial is to identify the feasibility and acceptability of personalized N-of-1 designs rather than to select the ideal treatment for each individual participant. Another potential limitation for the current trial is the lack of information about participants’ habitual melatonin levels before beginning the trial. Prior research suggests that individuals with lower endogenous melatonin secretion may benefit more from receiving melatonin replacement [64]. Therefore, the cause of participants’ self-reported poor sleep may not be influenced by melatonin.

Despite these potential limitations, this trial represents a significant step forward in the design of digital, remote, personalized trials. If it is found to be feasible and acceptable among individuals dealing with poor sleep, revision of the design of the intervention to evaluate alternative formulations of the current treatment (such as longer dose durations or different strength doses of melatonin) or multiple alternative treatments (such as behavioral interventions for sleep hygiene and exercise) in comparison to melatonin is possible. Further, if these personalized trials are found to be feasible as prior series of trials have been [47], we will be able adapt the platform used in this intervention for other health conditions and hopefully integrate personalized N-of-1 trials into clinical practice.

## Appendix

Multimedia Appendix 1 Insomnia Symptom Questionnaire.

Multimedia Appendix 2 Study Consent Form.

## Abbreviations

CSD: Consensus Sleep Diary
DSMB: Data and Safety Monitoring Board
EMA: ecological momentary assessment
GLS: generalized least squares
HIPAA: Health Insurance Portability and Accountability Act
IRB: institutional review board
ISI: Insomnia Severity Index
ISQ: Insomnia Symptom Questionnaire
SCN: suprachiasmatic nucleus
SUS: System Usability Scale

## Data Availability

Data archives containing the raw study data and data dictionaries for this study are available on the Open Science Framework platform [65].

## Dissemination

The trial results will be published in a peer-reviewed journal. All publications resulting from this series of personalized trials will follow the CONSORT extension for N-of-1 trials (CENT 2015) reporting guidelines [66]. Trial results will be reported to study collaborators and participants after study completion.
