# Supplementary material for: A Series of Remote Melatonin Supplement Interventions for Poor Sleep: Protocol for a Feasibility Pilot Study for a Series of Personalized (N-of-1) Trials
Source: JMIR Res Protoc. 2023 Aug 3;12:e45313. doi: 10.2196/45313 (PMC10436115; doi:10.2196/45313)
Supplement: Multimedia Appendix 1 [file resprot_v12i1e45313_app1.docx]

## **Multimedia Appendix 1**

## Insomnia Symptom Questionnaire

**Instructions:** If you have experienced **any** sleep symptoms **during the past month** please select the appropriate number to let us know how your sleep is affecting your daily life.

During the past month did you have…

Never 0 | Do not Know 1 | Rarely (less than once per week) 2 | Sometimes 1-2 times per week 3 | Frequently 3-4 times per week 4 | Always 5-7 times per week 5 | How long has the symptom lasted? (# of weeks, months, or years) [text box]

1. Difficulty falling asleep?
2. Difficulty staying asleep?
3. Frequent awakenings from sleep?
4. Feeling that your sleep is not sound?
5. Feeling that your sleep is unrefreshing?

**Instructions:** If you have experienced **any** sleep symptoms **during the past month** please select the appropriate number to let us know how your sleep is affecting your daily life.

During the past month…

Not at all 0 | A little bit 1 | Moderately 2 | Quite a bit 3 | Extremely 4

1. How much do your sleep problems bother you?
2. Have your sleep difficulties affected your work?
3. Have your sleep difficulties affected your social life?
4. Have your sleep difficulties affected other important parts of your life?
5. Have your sleep difficulties made you feel irritable?
6. Have your sleep problems caused you to have trouble concentrating?
7. Have your sleep difficulties made you feel fatigued?
8. How sleepy do you feel during the day?
9. How did you hear about this study? Please check all that apply.
   - - Email Listserv
     - Flyer
     - Internet Advertisement
     - Internet Search
     - Medical Clinic
     - Northwell e-mail blast
     - Northwell Health Research Portal
     - Past Personalized Trial participant
     - Personalized Trials Website
     - Social Media
     - Word of Mouth
     - Other

If “Other”, please tell us how you heard about this Personalized Trial: [text box]
