# Supplementary material for: A Series of Remote Melatonin Supplement Interventions for Poor Sleep: Protocol for a Feasibility Pilot Study for a Series of Personalized (N-of-1) Trials
Source: JMIR Res Protoc. 2023 Aug 3;12:e45313. doi: 10.2196/45313 (PMC10436115; doi:10.2196/45313)
Supplement: Multimedia Appendix 2 [file resprot_v12i1e45313_app2.docx]

**Multimedia Appendix 2**

**Northwell Health**

**Consent for Participation in a Research Study**

**Study Title:** Feasibility Study of Personalized Trials to Improve Sleep Quality

**Principal Investigator:** Karina W. Davidson, PhD, MASc

**Sponsor:** National Institutes of Health (NIH)

**Protocol Number: 21-0239-MRB**

**Consent Version Date: 9/27/22**

**About this research**

You are being asked to participate in a research study pilot.

This consent form will give you information about the study to help you decide whether you want to participate. Please read this form, and ask any questions you have, before agreeing to be in the study.

**Important Information**

This information gives you an overview of the research. More information about these topics may be found in the pages that follow.

| **Why am I being asked to provide my consent?** | This is a pilot research study, which is different than personal medical care.  Scientists do research to answer important questions which might help change or improve the way we do things in the future. |
| --- | --- |
| **Do I have to join this research study?** | No. Taking part in this pilot research study is voluntary.  You may choose not to take part in the pilot study or may choose to leave the pilot study at any time.  Deciding not to participate, or deciding to leave the pilot study later, will not result in any penalty or loss of benefits to which you are entitled.  This study may enroll employees of Northwell Health. Employee participation or non-participation will have no bearing on an employee’s position at Northwell Health.  This study may also enroll students, including those that may attend Hofstra University. Student participation or non-participation will have no bearing on a student’s grades or academic standing. |
| \| **Why is this research study being done?** \|  \| \| --- \| --- \| | The main purpose of this pilot study is to assess the feasibility and satisfaction of methods used for Personalized Trials (meaning your own personal response to N-of-1), including:   - Methods used to remotely recruit and enroll participants, - Using online web and social media advertising to recruit and enroll participants in research study - Methods used to collect and assess symptoms in response to a health and wellness strategy (in this case, the use of smart pill bottles, or medication adherence devices, and dietary supplements for sleep quality).   Our main goal is to see if an N-of-1 study design, or what we are terming Personalized Trials, can have widespread use in future research and clinical practice. We are not testing the safety or the effectiveness of the dietary supplements used in this study. |
| **What will happen to me during the study?** | In this pilot study, you will evaluate your response to two different doses of melatonin for sleep quality issues. Some weeks, you will take melatonin at 3 milligrams (mg), and some weeks you will take melatonin at 0.5 milligrams (mg). Other weeks you will be instructed to take a placebo pill, a pill that looks like the melatonin but has no effect (like a sugar pill). These supplements will be shipped to your address or an address of choice at no cost to you. You will not know when you are taking either of the doses of melatonin or the placebo, however the research team will now (this is called “single blinded”). Instead, these supplements will be marked in pill bottles labeled as “A”, “B”, or “C”. These pill bottles will be “smart” bottles, which will record when you take a pill from each bottle. During the study, you will be asked to manage your sleep as you normally would while refraining from taking any other melatonin or sleep aid medications during this study. Each day you will answer questions about your sleep and some questions about your pain, stress, fatigue, mood, confidence, and concentration. Every other week you will answer questions about your symptoms during the previous two weeks. During intervention periods, you will receive a text message each evening telling you which smart pill bottle to take a pill from. You will also wear a Fitbit activity monitor each day and night to track your daily activity and sleep. You will receive a participant report at the end of the study that tells you when you took 3 mg melatonin, 0.5 mg melatonin, or a placebo, and summarizes your observed data while you took each supplement or placebo. At the end of the study, we will ask you to complete a satisfaction survey and to participate in an audio-recorded virtual follow-up interview with a research coordinator to share your opinions about your Personalized Trials experience. After completing the 14 weeks of the study and the satisfaction survey and returning the smart pill bottles and charger to the study team (at no cost to you), you will be compensated $100 via a pay card (ClinCard). |
| **How long will I participate?** | The Personalized Trial will take place over the course of 14 weeks. You will be asked to complete a satisfaction survey after you receive your summary results (within 3 months of the Personalized Trial being complete). You will also be invited to participate in a 1-hour virtual follow-up interview to talk about your opinions about your experience. Study participation will end upon completion of the satisfaction survey. |
| **Will taking part expose me to risks?** | Some people may experience mild side effects from melatonin. These side effects could include drowsiness, daytime sleepiness, headaches, and nausea. Melatonin also has the potential to cause a “hangover effect” in the form of daytime sleepiness or drowsiness. These side effects normally go away quickly on their own. All of the known potential risks are listed in the risks section below. You will be instructed to take the supplement pill one hour before your usual bedtime, and you should neither drive nor operate heavy machinery within 5 hours after taking the nightly supplement.  Another risk of taking part in this pilot study is the possibility of a loss of confidentiality or privacy. Some of the questions we ask in the survey are personal. You may feel embarrassed or stressed. You could also experience mild skin irritation (rash) from wearing the Fitbit activity monitor. |
| **Are there any benefits to participation?** | A Personalized Trial to Improve Sleep Quality is not designed to benefit you directly. You may or may not experience any effects in the moment when taking either dosage of melatonin. However, in receiving your observed data trends when taking either supplement (in the participant report), you will find out information about your own personal response to melatonin which may give you information on how to manage your sleep quality. We cannot promise any benefits to others from your taking part in this research. However, possible benefits to others include demonstrating that a Personalized Trials design can help doctors and scientists discover new ways to help patients in the future. |
| **What are my alternatives to participation?** | Since this project is assessing the Personalized Trial study design, the alternative is not to participate. |

**Please review the rest of this document for details about these topics and additional things you should know before making a decision about whether to participate in this research. You should ask questions before you decide if you want to participate. You can also ask questions at any time during the study: personalizedtrials@northwell.edu**

**Introduction and Research Purpose:**

You are being asked to join a pilot research study. The purpose of this research study is to assess the feasibility and satisfaction of methods used for Personalized Trials, including:

- Methods used to remotely recruit and enroll participants,
- Using online web and social media advertising and methods to recruit and enroll participants in research study
- Methods used to collect and assess symptoms in response to a health and wellness strategy (in this case, the use of smart pill bottles, or medication adherence devices, and dietary supplements for sleep quality).

This data will be used to inform future Personalized Trials, and it will help us understand the needs for a future technology platform to conduct these trials. We are testing these methods and technology to help determine if a Personalized Trial design, or a research study that occurs in only one patient, can have widespread use in future research and clinical sessions.

This particular pilot study uses a Personalized Trials methodology to see if it can be useful in observing trends in melatonin for poor sleep quality. We are not testing the safety or the effectiveness of the melatonin. You are being asked to participate in this pilot study because you have been identified as having poor sleep quality for at least the last month.

You do not have to be in this pilot study to receive medical care. You should ask questions before you decide if you want to participate. You can also ask questions at any time during the pilot study by emailing [personalizedtrials@northwell.edu](mailto:personalizedtrials@northwell.edu) or calling (646) 864-6020.

**Why am I being asked to participate?**

You are being asked to participate in this study because you have self-reported poor sleep quality.

**How many people will take part in this study?**

This pilot research study hopes to enroll up to 150 people. Up to 60 participants will complete research procedures in a randomized order (like the flip of a coin).

**How long will you be in this study?**

If you are selected to take part in this pilot study, the study procedures will last for 14 weeks.

If you will be traveling outside of the United States during the pilot study period, or if you will not have access to text-messaging or internet for more than a few days during the pilot study period, you should talk to a member of the study team to determine your eligibility for this study.

**What will happen in this research study?**

After filling out this consent form, you will be asked to provide more information about yourself. This will include your cell phone carrier and home address. This pilot study uses text-message reminders to help you through the study protocol. Receiving the text messages will require cellular data and completing the surveys will require cellular data if you are not connected to Wi-Fi. You will not be reimbursed for text messages or data charges, and standard carrier rates may apply.

If you are selected as a potential research participant in this pilot research study, you will be sent your first study kit. This will include a Fitbit device. Additional materials to help guide you through this pilot study will be sent by mail or electronically. You will be sent a text to confirm your pilot study start date, and you will also receive text messages ahead of when your study begins to prepare you for your study start date. You will be asked to download the Fitbit app to your phone to collect your data. Correspondence sent by the study team will reference “the sleep quality study” or “the personalized trial to improve sleep quality.” Other than the reference to the study title, e-mail correspondence from the study team will not contain protected health information. Communications sent over the n1Thrive platform are encrypted, which means that others will be unable to access the information transmitted.

The first two weeks of the study is considered your baseline period. The purpose of this baseline period is to help us determine your usual activity and symptoms without any use of melatonin for your sleep quality. You will not receive any melatonin during your baseline period, and you will be asked to refrain from taking any melatonin or other sleep aid medication of your own. Each morning you will receive a survey asking you a few questions about your previous night’s sleep. This survey will take approximately 5 minutes to complete. At three random times each day, you will receive a text message asking you to rate your pain, fatigue, stress, mood, confidence, and concentration levels at that exact moment. This survey will take approximately three minutes of your time. On every other Sunday evening, at the end of each intervention block, you will receive a slightly longer survey asking you to reflect on your sleep quality during that intervention block. This survey will take less than 10 minutes to complete.

You will be asked to wear your Fitbit all day and night, especially while you are sleeping. You will be asked to charge your Fitbit device at least every four days. Your Fitbit can be charged during periods of extended sitting, like when you are in your car, sitting at a desk, or while showering. At least every two days you will need to sync your Fitbit device. You can sync your device by opening the Fitbit app and waiting for your data to load. You will receive a text message reminder asking you to sync your Fitbit if you have not done so in 48 hours, and you will receive a reminder asking you to charge your device when the battery is approaching being empty.

It is very important that you wear your Fitbit device all day and night, that you sync your

Fitbit device at least every two days, that you charge your Fitbit device at least every four days, and that you answer the survey questions sent to you each day. Completing these requirements is often referred to as adherence or compliance and is very crucial in the study. You will be able to select the best times to receive your surveys and reminders before your baseline period begins, and you will have the opportunity to change these times every other week if there is a change in your schedule. You may be asked to discontinue your participation in this study if you are not wearing your Fitbit long enough, or if you do not answer enough survey questions each day.

If satisfactory adherence completion is achieved during your baseline period, you will be contacted by a member of our pilot study team by text with your intervention schedule for the rest of the study. You will be assigned a random order of 6 intervention periods. Each intervention period will last 2 weeks. An intervention period may consist of taking 3 mg melatonin, 0.5 mg melatonin, or a placebo. You will not know when you are taking 3 mg melatonin, 0.5 mg melatonin or a placebo, but the research team will know (this is called "single blinded”). Instead, you will be mailed a second study kit, which will include these pills inside three smart pill bottles marked “A”, “B”, and “C”. These smart pill bottles are often called medication adherence devices, and they will record each time a pill is taken from the bottle. You will be sent instructions on how to activate the devices. These devices will be linked to your de-identified study ID by a member of our research team. Nomi by SMRxT will not receive any identifying information about you. We will use the date and time stamps from these smart pill bottles to track your progress through the study. You will also receive a schedule and text message reminders that tell you when you are assigned to take a pill from bottle “A”, when you are assigned to take a pill from bottle “B”, and when you are assigned to take a pill from bottle “C”. If we see that you are not using the pills/pill bottles appropriately, we will contact you to remind you of your assigned intervention schedule.

During intervention weeks, you will be instructed to take one pill from one assigned smart pill bottle approximately one hour before your usual bedtime. You should not drive or operate heavy machinery for 5 hours after taking the pill. Your study calendar will tell you which smart pill bottle you should use each week. You will also receive a text message to remind you which smart pill bottle to use each night. Each smart pill bottle will have four weeks’ worth of pills in them. You will be assigned to one smart pill bottle in a single intervention period before switching to a new smart pill bottle. Each intervention period will last 2 weeks. On Sunday evenings, one day before you switch which pill you take, you will receive a text message alerting you to that change.

During intervention weeks, you will also be asked to continue wearing your Fitbit device each day and night. You will continue to receive 4 to 5 text messages every day with survey questions about your sleep, pain, fatigue, stress, mood, concentration, and confidence levels. You will also be asked about any pill-related side effects you may be experiencing. If you are concerned about any side effects, you may stop taking the pills at any time and contact a member of the research team for more information about continuing your study participation. Additionally, you will receive a text message reminding you which smart pill bottle to take a pill from each night at a preferred time. This time can be changed throughout the study. You will also continue to receive a slightly longer biweekly survey at the end of each intervention block reflecting on your sleep from the last two weeks.

During the pilot study period, you may also receive additional text messages to remind you of next steps for this study or with reminders about study protocol. We will send a maximum of 5 text message surveys per day during the study, as well as these reminders and the survey questions outlined above.

The intervention portion of the study will end once you have completed one baseline period (two weeks), two periods of melatonin at 3 mg (two weeks each, four weeks total), two periods of melatonin at 0.5 mg (two weeks each, four weeks total), and two periods of placebo (two weeks each, four weeks total), or 14 weeks total.

Your participation in this pilot research study is voluntary. You may stop participating at any time by the methods described in the relevant section below. Alternatively, you may be asked to end your study participation by a member of our research team for any reason.

We will ask you to return, at no cost to you, the 3 smart pill bottles and the device charger at the end of the study. The smart pill bottles and the device charger can be placed in the provided padded envelope and sent back to the research team at no charge to you. This envelope will have a prepaid label on it and will be sent to you along with shipping instructions.

We will compile the data from your questionnaires, the smart pill bottles, and your Fitbit. Any identifying information about you will be removed. A statistician will then analyze your coded data. Only the research team will have the key to identify you based on your research code. We will then turn this analysis into a report of your observed data and symptoms over the study period. You will be sent this report in a secure message by our study team. This report is not meant to offer medical advice, but you may find it useful for your own knowledge because it could help you understand your personal response to each dose of melatonin. You will be sent a satisfaction survey within one week of receiving your report.

After being sent your report, you will be asked to complete a satisfaction survey and invited to participate in a virtual follow-up interview (such as a video call over Microsoft Teams) with a member of our study team to talk about your experience as a research participant in this pilot study. We will ask you several questions about what it was like to participate in the study. We will also ask about your opinion on Personalized Trials. This interview will be audio-recorded and transcribed for data collection purposes only; however, if you decline to be audio recorded, you may still participate in the follow-up interview.

Your participation in the pilot study is complete after you completed 14 weeks of data collection, the study team has received back the 3 smart pill bottles and charger assigned to you, and you have completed your satisfaction survey. Upon full completion of the pilot study, you will receive a $100 ClinCard as a thank you for your participation. You can use this card like a credit or debit card anywhere MasterCard is accepted, including online.

**What are the risks of the research study? What could go wrong?**

There may be risks or discomforts if you take part in this study, including mild side effects from taking melatonin such as drowsiness, daytime sleepiness, headaches, and nausea. Melatonin also has the potential to cause a “hangover effect” in the form of daytime sleepiness or drowsiness.

There are some other potential side effects of taking melatonin that you should know about. These side effects are less common than the ones listed above, but there is a chance that you could experience them, although that chance is low. Less common side effects include short-lasting feelings of depression, mild tremor, mild anxiety, abdominal cramps, irritability, reduced alertness, confusion or disorientation, and abnormally low blood pressure. These side effects have typically only been observed at doses higher or durations longer than the melatonin you will be taking during this pilot study.

You should not drive nor operate heavy machinery for 5 hours after taking your nightly supplement during this pilot study. The nightly supplement will be administered one hour before bedtime, so these side effects should pose minimal disruption and risk.

If you are concerned about any of these side effects or any others you may be experiencing, you should immediately stop taking the melatonin and contact a member of the study team. You may still be able to continue with the study with modified melatonin intake, or you may be asked to end your participation in the study.

You may also experience mild skin irritation (rash) from wearing the Fitbit band during this research study. To reduce irritation, keep the band clean and dry. To provide relief for you skin if this mild risk occurs, remove the band for a short period of time.

In addition, another potential risk of taking part in this study is the possibility of a loss of confidentiality or privacy. Loss of privacy means having your personal information shared with someone who is not on the study team and was not supposed to see or know about your information. The study team plans to protect your privacy by only sharing necessary information about you to those outlined in the “Who else will see your information?” section below.

Lastly, some of the questions we will ask you are personal. You may feel embarrassed or stressed. You may ask to see the questions before deciding whether or not to take part in this study.

**What are the benefits of this research study?**

This Personalized Trial to Improve Sleep Quality is not designed to benefit you directly. You may or may not experience any effects when taking melatonin, but in receiving your observed data trends when taking either dose of melatonin, you will find out information about your own personal response to melatonin which may give you information on how to manage your sleep quality. We cannot promise any benefits to others from your taking part in this research. However, possible benefits to others include demonstrating that a Personalized Trial design can help doctors and scientists discover new ways to help patients in the future.

**Will I receive my results?**
Although the main purpose of this pilot study is to assess the methods for future Personalized Trials, the data we collect from you during study activities could impact how you treat your poor sleep quality. For this reason, much of the information collected about you during the pilot study will be analyzed and provided back to you. You will receive this data in the form of a personalized participant report. The data included in the participant report will include graphs and statistics about your self-reported sleep quality; self-reported pain, stress, fatigue, mood, confidence, and concentration; activity patterns; sleep patterns; and adherence (i.e., how closely you followed the study procedures). These data will be shown in relation to weeks you had melatonin at 3 mg, weeks you had melatonin 0.5 mg, and weeks you had the placebo. This report is meant to summarize your observed data and self-reported symptoms and should not be considered medical advice.

**If you do not want to take part in this research study, what are your other choices?**

Since this project is assessing the Personalized Trial study design, the alternative is not to participate.

If you are interested in learning about effective interventions for your symptoms of poor sleep quality, or if you want to learn more about your personal results, you may wish to meet with professionals with expertise to help you learn more about available interventions. The study team/study will not cover the costs of any follow-up consultations or actions.

**Are there any costs for being in this research study?**

This pilot research study is funded by the National Institutes for Health (NIH). All study related equipment, devices, procedures, and supplements (i.e., melatonin) will be provided to you at no cost. Neither you nor your insurance company will be billed for your participation in this research.

This pilot study uses text messages to deliver notifications, reminders, and study questionnaires. Standard message and data rates from your wireless carrier may apply. You will not be compensated for any costs related to data usage or sending or receiving text messages by the study or by members of the study team.

**Will you receive any payments for participating in this research study?**

After successfully completing all aspects of this study, including completing data collection and your satisfaction survey, you will be mailed a $100 pay card called a ClinCard that can be used like a credit or debit card anywhere MasterCard is accepted, including online. As a thank you for participation, you will be able to keep your Fitbit device (a value of $149.95).

**What are your rights as a research participant?**
Your participation in this project is voluntary. The quality of your medical care will be the same, whether you join, refuse to join, or decide to leave the pilot study.

If you do not join the pilot study you will not be penalized or lose benefits to which you are entitled. If you join the study you may withdraw at any time without prejudice to your future care and/or employment at Northwell Health.

This study may enroll employees of Northwell Health. Employee participation or non-participation will have no bearing on your position at Northwell Health.

This study may also enroll students, including those that attend Hofstra University. Student participation or non-participation will have no bearing on your grades or standing at your academic institution.

**Could you be taken off the study before it is over?**

It is also possible that your participation in this pilot study may end without your consent. This decision may be made by a researcher, study sponsor or the Institutional Review Board (IRB- the committee that oversees research at this institution).

Reasons for withdrawal may include:

- Failure to follow the study protocol,
- Self-reported adverse side effects to two or more interventions
- Significant cell phone carrier issues that prevent you from receiving study text messages
- It is not in your best interest to continue this pilot study, or
- The pilot study is stopped.

If you withdraw from this pilot study or if you are withdrawn from the pilot study, any data already collected will continue to be used.  However, no new data will be collected.  

**What happens if new information is learned?**

You will be told of any new findings that may change your decision to continue to participate. Your consent to continue to take part in this pilot study may be obtained again.

**What information will be collected and used for this study?**

If you agree to be in this pilot study, we will collect information that identifies you. We may collect the results of questionnaires, interviews, Fitbit activity and sleep, smart pill bottle use, and video views. We will only collect information that is needed for the research. This information has been described in this consent form. If you sign this consent form, you are giving us permission to collect, use and share your health information. This permission is called authorization. Your authorization to use and share your health information will never expire. If you do not want to provide authorization, then you cannot participate in this research study.  Text messages will alert you to a new message from “N1Thrive” and contain a link to open this secure browser directly on your phone. No identifying information will be shared via text message. The study team will have direct access to the survey and Fitbit data, as applicable, through N1Thrive.

Data collected by Fitbit® includes activity data (steps, activities, intensity, breathing rate, heart rate, heart rate variability, floors climbed), sleep data (total sleep minutes, sleep stage estimates, sleep and wake times), and device data (last sync date and Fitbit battery level).

You will be asked to download the Fitbit app to your personal phone in order to use the Fitbit device. You will also be sent instructions on how to connect the device to a study account. General email addresses that have been created for this research study have been used to create these study accounts without any information that could identify you. It is important that you use the Fitbit account provided during the study to protect your information, and to allow us to collect your data. Your IP address may be collected by the Fitbit app which could be considered identifiable information. Your Fitbit data will be securely stored in Fitbit electronic platforms as is standard for all Fitbit users. Fitbit’s Terms of Service and Privacy Policy are separate from this research consent form, but should be considered before you sign this consent from. To join this study, a Fitbit account will be created for you. Study staff will agree to the [Fitbit Terms of Service](https://www.fitbit.com/global/us/legal/terms-of-service) (https://www.fitbit.com/global/us/legal/terms-of-service) and [Privacy Policy](https://www.fitbit.com/global/us/legal/privacy-policy) (https://www.fitbit.com/global/us/legal/privacy-policy ) when they create your account using a study specific username and password.  Fitbit’s Terms of Service includes information about your legal rights when using Fitbit’s products that may differ from your rights as a participant in this study.  Fitbit’s Privacy Policy describes how Fitbit collects, uses, shares, and protects your data. You can exercise your right to access your personal information by logging into your Fitbit account and using your account settings. Fitbit may also have access to device identifiers so they may be able to identify that you are a participant in this research. For more information about the information that Fitbit may have access to, refer to Fitbit’s Privacy Policy.  No information that can be used to identify you will be associated with your Fitbit study account. We will only collect this data through the 14-week pilot study period. Once your study period is complete, you will be sent instructions on how to un-link your Fitbit device from the account. Data from your Fitbit device may be shared with Northwell Health for research purposes through N1Thrive or an online portal called Fitabase. The study account given to you to connect your Fitbit will be linked to an identification number in the Fitabase system. No information that could be used to identify you will ever be shared with Fitabase. Only the research team will have access to data that will be able to connect a research participant to their Fitabase ID. Fitabase will stop sharing your data at the end of your study, but as an added step, you will be asked to remove the study account from your device if you would like to keep your Fitbit. Data collected by Nomi includes date and timestamps of when the smart pill bottles were opened, and the weight of the smart pill bottle’s contents throughout the study. The smart pill bottles will cease activity at the end of the intervention period. Once your study period is complete, you will be sent instructions on how to return your Nomi smart pill bottles and charger using the pre-paid shipping supplies included in your study materials. No personal data is collected or stored on the Nomi device. All stored date, time, and weight stamps on the bottle will be wiped from the devices upon successful return to the manufacturer.

The follow-up interview will be audio-recorded and transcribed for data collection purposes only. Audio recordings will be destroyed upon completion of the study.Survey data will be collected via a secure web browser and stored in a HIPAA-compliant, Northwell approved database called N1Thrive, the company the study team is partnering with to develop the technology to analyze data for current and future Personalized Trials. Secure text messages will alert you to a new message from “N1Thrive” and contain a link to open this secure browser directly on your phone. No identifying information will be shared via text message. The study team will have direct access to the data shared through N1Thrive.

**Who else will see your information?**

Study records that identify you will be kept private. You will not be identified in study records or publications disclosed outside Northwell Health, except as detailed below.

Investigators will share information collected from this pilot research study with:

- Study sponsor (NIH) and/or its agents,
- Other researchers,
- Accrediting agencies,
- Data safety monitoring board,

 The following reviewers may access your study records to make sure that this study is being done properly:

- Representatives from Federal and state government oversight agencies, such as the Department of Health and Human Services, and the National Institutes of Health,
- Representatives from Northwell Health’s Human Research Protection Program (a group of people that oversee research at this institution)

We will do our best to protect the privacy of your records but it is possible that once information is shared with people listed on this form, it may be released to others. If this happens, your information may no longer be protected by the federal law. In the future, we may publish results of this pilot study in scientific journals and may present it at scientific meetings. If we do, we will not identify you.   If the researchers learn about potential serious harm to you or someone else or other public health concerns, it will be shared with the appropriate authorities.

If you agree to let us use and disclose your protected health information, we will collect your health information until the end of the research.

**Can you change your mind?**

If you change your mind about being in the pilot study, you may withdraw at any time. If you want us to stop collecting your health information, you need to send an email to the researcher at the following address: [personalizedtrials@northwell.edu](mailto:personalizedtrials@northwell.edu). Alternatively, you can send a letter to the researcher at the following address:

Dr. Karina W. Davidson 
Center for Personalized Health

130 East 59^th^ Street, Suite 14C

New York, NY 10022

Your email or letter needs to say that you have changed your mind and do not want the researcher to collect and share your health information. You may also need to leave the pilot research study if we cannot collect any more health information. We may still use the information we have already collected. We need to know what happens to everyone who starts a research study, not just those people who finish it.

**Will information about this study be available to the public?**

A description of this clinical trial will be available on http://www.Clinical Trials.gov , as required by U.S. Law. This Web site will not include information that can identify you. At most, the Web site will include a summary of the results. You can search this website at any time. In addition, the researcher also plans to share information about the pilot study, including de-identified data, on the following data sharing website: https://cos.io/. The Open Science Framework is a free, open-source web application built to provide researchers with a free platform for data and materials sharing. There will be no identifiable data posted to this website or used in future studies.

**Certificate of Confidentiality**

To help us protect your privacy, this research is covered by a Certificate of Confidentiality from the US Department of Health and Human Services (DHHS). The Certificate of Confidentiality means that researchers cannot be forced to identify you, even under a court subpoena. The Certificate does not mean the Secretary of DHHS approves or disapproves of the project. It adds special protection for the research information about you. You should know, however, that researchers may provide information to appropriate individuals or agencies if harm to you, harm to others or child abuse becomes a concern. A Certificate of Confidentiality does not prevent you or a member of your family from voluntarily releasing information about yourself or your involvement in this research. However, if an insurer, employer or other person learns about your participation and gets your consent to receive research information, then the researchers will have to provide your information.

**Will my information be used for research in the future?**
Information collected from you for this research may be used for future research studies, pooled with other personalized trial or CPH participants or shared with other researchers for future research. If this happens, information which could identify you will be removed before any information is shared. Since identifying information will be removed, there will not be an additional consent for future research. By consenting to participate in this study you are agreeing to allow your data to be used by future researchers without additional consent. This information will be stored on a secure database. It will only be accessible by trained members of the study team. If you change your mind about being contacted in the future, you may follow the procedures outlined above to notify the researcher.

**Does the investigator of this study receive money if you take part?**

The investigators on this pilot study receive money to conduct the study, but do not financially benefit from your participation. The money they receive is to pay them back for the costs of conducting the research study. Funding for this research study is provided by the National Institutes of Health (NIH)*.*

**Who can answer your questions about this study?**

If you have any questions about the pilot study, you may call Alexandra Perrin, Clinical Research Coordinator, 646-864-6020 or email [personalizedtrials@northwell.edu](mailto:personalizedtrials@northwell.edu). If you have questions about side effects or injury caused by research you should call Joan Duer-Hefele RN, MA, CCRC, Director of Clinical Research, at (646) 766-7153. If you need emergency care, dial 911 or go to the nearest Emergency Room. If you have questions about your rights as a research participant, concerns about being in the study, or would like to offer input, you may contact the Office of the Institutional Review Board (the committee that oversees research at this institution) at (516) 465-1910.

A signed copy of this consent form will be shared with you.

[Signature Page Follows]

**Please respond to the following questions to demonstrate your understanding of study procedures and your rights as a research participant.**

1. As a participant, I will need to wear my Fitbit 24 hours a day, even while I am sleeping.
   - True
   - False
2. As a participant, I will be instructed to take one pill from an assigned smart bottle, 1 hour before my usual bedtime every night during the pilot study (after I successfully pass baseline).
   - True
   - False
3. As a participant, I can remove myself from the pilot study at any time by contacting the researcher.
   - True
   - False
4. As a participant, I will receive at most 5 text message surveys per day, along with other important text message communications.
   - True
   - False

[Signature Page Follows]

**Summation/Signature**

You have read the above description of the research study. You have been told of the risks and benefits involved and all your questions have been answered to your satisfaction. A member of the research team will answer any future questions you may have. You voluntarily agree to join this study and know that you can withdraw from the study at any time without penalty. By signing this form, you have not given up any of your legal rights.

____________________________________________________________

Printed Name of Participant

___________________________________________________ ______________

Signature of Participant Date
